# Antioxidant and cytoprotective activities of *Piper betle, Areca catechu, Uncaria gambir* and betel quid with and without calcium hydroxide

**DOI:** 10.1186/1472-6882-13-351

**Published:** 2013-12-11

**Authors:** Nordin Nur Sazwi, Thurairajah Nalina, Zubaidah Haji Abdul Rahim

**Affiliations:** 1 Department of Oral Biology, Faculty of Dentistry, University of Malaya, 50603 Kuala Lumpur, Malaysia; 2 UCSI University, #1, Jalan Menara Gading, UCSI Heights, Cheras, 56000 Kuala Lumpur, Malaysia

**Keywords:** *Piper betle*, *Areca catechu*, *Uncaria gambir*, Calcium hydroxide, Betel quid, Antioxidant, Cytoprotective, LC-MS/MS

## Abstract

**Background:**

Betel quid chewing is a popular habit in Southeast Asia. It is believed that chewing betel quid could reduce stress, strengthen teeth and maintain oral hygiene. The aim of this study was to investigate the antioxidant and cytoprotective activities of each of the ingredients of betel quid and compared with betel quid itself (with and without calcium hydroxide). The correlation of their cytoprotective and antioxidant activities with phenolic content was also determined.

**Methods:**

Five samples (betel leaf, areca nut, gambir, betel quid and betel quid containing calcium hydroxide) were extracted in deionized distilled water for 12 hours at 37°C. Antioxidant activities were evaluated for radical scavenging activity using DPPH assay, ferric reducing activity using FRAP assay and lipid peroxidation inhibition activity using FTC assay. Total phenolic content (TPC) was determined using Folin-Ciocalteu procedure. Phenolic composition was analyzed using LC-MS/MS. Cytoprotective activity towards human gingival fibroblast cells was examined using MTT assay.

**Results:**

Among the ingredients of betel quid, gambir demonstrated the highest antioxidant (DPPH - IC_50_ = 6.4 ± 0.8 μg/mL, FRAP - 5717.8 ± 537.6 μmol Fe(II)/mg), total phenolic content (TPC - 1142.5 ± 106.8 μg TAE/mg) and cytoprotective (100.1 ± 4.6%) activities. Betel quid when compared with betel quid containing calcium hydroxide has higher antioxidant (DPPH - IC_50_ =59.4 ± 4.4 μg/mL, FRAP - 1022.2 ± 235.7 μmol Fe(II)/mg), total phenolic content (TPC - 140.0 ± 22.3 μg TAE/mg), and cytoprotective (113.5 ± 15.9%) activities. However, all of the five samples showed good lipid peroxidation inhibition compared to vitamin E. LC-MS/MS analysis revealed the presence of quinic acid as the major compound of gambir and betel quid. A positive correlation was observed between TPC and radical scavenging (r = 0.972), reducing power (r = 0.981) and cytoprotective activity (r = 0.682).

**Conclusions:**

The betel quid has higher TPC, and antioxidant and cytoprotective activities than betel quid with calcium hydroxide. The quinic acid in betel quid may play an important role in the oral health protection.

## Background

It has been reported that betel leaf, areca nut and gambir have a wide spectrum of therapeutic properties. The betel leaf has antiplaque [1], anti-diabetic and anti-inflammatory properties [2,3] as well as anti-proliferative effect towards KB and HeLa cells [4]. Areca nut possesses significant analgesic and anti-inflammatory [5], wound healing [6] antidepressant [7] and anti-HIV activities [8]. The gambir has been used for the treatment of diarrhea, dysentery and as an astringent medicine in Asian countries [9]. Generally plants that have significant therapeutic properties were found to be rich in phenolics and have high antioxidant properties. This correlation has been confirmed with the antioxidant activity being detected in the extract of betel leaf [10] areca nut [11] and gambir [12].

The consumption of antioxidant-rich foods will help to neutralize the free radicals in the body, thus preventing or delaying the oxidative damage of lipids, proteins and nucleic acids [13]. It has been shown that the antioxidants could reduce mortality rate of cardiovascular disease [14,15], and protect against cancer and other chronic diseases [16]. In addition, many studies have reported that the antioxidant is highly associated with the cytoprotective activity of plant [17,18]. However, the plant antioxidant compounds that are responsible for reducing the risk of chronic disease have not been identified [19].

In Malaysia, the betel quid usually consists of dried areca nut, gambir and calcium hydroxide wrapped in betel leaf (personal communication with the local community in Gombak, Kuala Lumpur). The nut of *Areca catechu* is usually sun-dried and cut into pieces. Gambir is prepared by boiling the leaves and barks of *Uncaria gambir* of the family Rubiaceae in water. The liquid extracted from the plant are dried and molded into large size pellet. Calcium hydroxide (also known as slaked lime) is usually made from burnt and crushed seashell.

It has been claimed that chewing betel quid could strengthen teeth and gums and maintain oral hygiene [20]. Betel quid chewing has been practiced for centuries and it is the fourth typical habit in the world after nicotine, alcohol and caffeine consumption [21] with an estimate of 600 million betel quid chewers worldwide [22]. This habit is popular among the elderly and lower income population in rural areas, primarily in Southeast Asia and has become part of their social and cultural practices [23]. Betel quid is chewed to reduce stress, increase alertness and produce feelings of psychological well-being [24,25]. It gives a warm and bitter taste with aroma, and produces red juice, which would stain teeth red.

At present, there are limited researches on antioxidant and cytoprotective activities of betel quid. Hence in this study, the antioxidant and cytoprotective activities of betel quid (with and without calcium hydroxide) were evaluated and compared with those of its respective ingredients. The correlation of the antioxidant and cytoprotective activities with phenolic content was also determined.

## Methods

### Chemicals and reagents

Folin-Ciocalteu’s phenol reagent and ferric chloride tetrahydride were purchased from MERCK (Germany). Ethanol and ascorbic acid were purchased from Fisher Scientific (USA). Hydrochloric acid, monosodium phosphate and disodium phosphate were purchased from R & M Chemicals (UK). Alpha-tocopherol, linoleic acid, DPPH (1,1- diphenyl-2-picrylhydrazyl) and TPTZ (2,4,6- tripyridy-s-triazine) were purchased from Sigma-Aldrich Inc. (USA). Tannic acid was purchased from Bendosen (UK). Dulbecco’s modified eagle medium (DMEM) (11960-044) and fetal bovine serum (10270-098) were purchased from Gibco BRL (USA). Ammonium thiocyanate, phosphate buffer saline, gelatine (from bovine skin), dimethyl sulfoxide (DMSO), 3-(4,5-dimethylthiazole-2-yl)-2,5-diphenyl-tetrazoliumbromide (MTT) were purchased from Sigma-Aldrich Inc. (USA). Penicillin/streptomycin (100×), fungizone (Amphotericin B, 250 μg/mL), acutase cell detachment solution were purchased from PAA (UK).

### Sample collection

Fresh betel leaves (*Piper betle*) were purchased fresh from one source in Keramat, Kuala Lumpur. The herbarium voucher specimen was authenticated and identified by the morphology and fragrance of the leaves by Mr Kamarudin Saleh from the Herbarium Unit of the Forest Research Institute Malaysia (FRIM, Kepong, Selangor, Malaysia). The betel leaves were prepared and deposited according to the regulation specified by the FRIM Herbarium. The voucher number for *Piper betle* leaves is PID 411113-20.

The processed form of gambir and the dried form of areca nut and calcium hydroxide were purchased from a shop at the Chow Kit market in Kuala Lumpur, Malaysia and all were purchased at three different times (in order to prepare three sets of extractions) started from June to December 2010.

### Preparation of the aqueous extract of betel quid (with and without calcium hydroxide) gambir, areca nut and betel leaf

A 10% aqueous sample extract was prepared according to the following procedure. A known weight of betel leaf was homogenized in deionized distilled water that was placed in an incubator shaker at 37°C for 12 hours. This was followed by filtration using No.1 filter paper (Whatman, USA) before concentrating using a rotary evaporator. The concentrated extract was then freeze-dried and stored at -20°C until further use. The procedure was repeated with areca nut, gambir, betel quid and betel quid containing calcium hydroxide. The betel quid was prepared using betel leaves, areca nut and gambir in the proportion of 46:3:1. The betel quid with calcium hydroxide was prepared using the proportion 43:3:1:3 for betel leaves: areca nut: gambir: calcium hydroxide respectively. This is the proportion that is popularly consumed by the locals in Malaysia (personal communication). For each of the samples, three different extract preparations were carried out. pH of the respective extracts were measured and recorded prior use.

### DPPH radical scavenging activity

The radical-scavenging activity of the extracts against 1,1-diphenyl-2-picrylhydrazyl (DPPH) was assessed using the method described by Manigauha and Maheshwari [26]. Ascorbic acid was used as a positive control while the DPPH-ethanol mixture without the extract was used as the blank control. The percentage inhibition of DPPH radical was calculated using the following formula:

$$\begin{array}{c} \mathrm{Inhibition}\;\mathrm{Percentage}=\left(A_{\mathrm{blank}\;\mathrm{control}}–A_{\mathrm{sample}}\right)\\ \;/A_{\mathrm{blank}\;\mathrm{control}}\times 100 \end{array}$$

where A _sample_ is the absorbance of extracts/ascorbic acid and A _blank_ control is the absorbance of the blank control. The antioxidant activity was expressed as IC_50_ value, which is defined as the amount of antioxidant that is required to decrease the initial DPPH concentration by 50%.

### Ferric reducing activity

The ferric reducing activity of the extracts was determined using the ferric reducing antioxidant power (FRAP) assay developed by Benzie and Strain [27]. The FRAP reagent was freshly prepared by mixing 10 mM TPTZ solution in 40 mM HCl, 20 mM FeCl3, 300 mM acetate buffer (pH 3.6) in proportions of 1:1:10 (v/v/v). Ten μL of extract (1 mg dissolved in 1 mL deionised distilled water) was dispensed into a 96- well microplate. Subsequently 200 μL of freshly prepared FRAP reagent that has been warmed at 37°C was dispensed into the same well of 96-well microplate containing the extract. The mixture was then mixed and the absorbance was measured after 4 min at 593 nm using a microplate reader (Power Wave-X340 Bio-tek instruments Inc.). Ferrous sulphate (concentrations ranging from 200 to 1000 μM, R^2^ = 0.9940) was used to plot the standard curve. Ascorbic acid was used as the reference. The FRAP was expressed as micromoles of ferrous equivalents, Fe(II) per mg of extract.

### Inhibition of lipid peroxidation activity

The inhibition of lipid peroxidation activity of the extracts was carried out using ferric thiocyanate (FTC) assay described by Osawa and Namiki [28]. To prepare a stock mixture of the extract, 4 mL of extract (50 μg/mL) in 95% (w/v) ethanol was added to 4.1 mL of linoleic acid (2.5%, v/v) in 99.5% ethanol, followed by 8 mL of 0.05 M phosphate buffer pH 7.0 and 3.9 mL of distilled water. The stock mixture was then kept in an incubator at 40°C in a dark bottle loosely capped until further use. For measurement of the antioxidant property of the extract, 0.1 mL of the stock mixture was added to a mixture containing 9.7 mL of 75% ethanol (w/v), 0.1 mL of 30% ammonium thiocyanate (w/v) and 0.1 mL of 20 mM ferrous chloride in 3.5% (v/v) hydrochloric acid. After exactly 3 min, the absorbance reading of the complex formed was measured at 500 nm and the reading was repeated at every 24 hours for eight days. For blank control, 4 mL of ethanol (95% w/v) and for positive control 4 mL of α-tocopherol (50 μg/mL) was added instead of the extract. The antioxidant activity was determined by calculating the percentage of lipid peroxidation inhibition using the formula:

$$\mathrm{Inhibition}\;\mathrm{Percentage}=\left(A_{\mathrm{control}}–A_{\mathrm{sample}}\right)/A_{\mathrm{control}}\times 100$$

### Determination of total phenolic content

Total phenolic content of the extracts was determined using modified version of Folin-Ciocalteu method described by Chandler and Dodds [29] and Shetty et al. [30]. One mg of each extract was respectively dissolved in 1 mL deionised distilled water and this was subsequently diluted with the addition of another 4 mL of the distilled water. This was followed by the addition of 0.5 mL of 50% Folin-Ciocalteu reagent. The mixture was then vortexed and left for 5 mins at room temperature. One mL of sodium carbonate (5% w/v) was then added and after mixing thoroughly, the mixture was incubated in the dark for 1 hour at room temperature, after which its absorbance was measured at 725 nm using UV/Vis spectrophotometer (Shimadzu UV-1800, Japan). Tannic acid (concentration ranging from 5 to 80 μg/mL, R^2^ = 0.9993) was used to plot the standard curve. The TPC was expressed as μg of tannic acid equivalents (TAE) per mg dried extract.

### Analysis of phenolic composition

The extracts (0.002 g/mL) were filtered using 0.2 mm nylon membrane filters prior to injection into the HPLC. The chromatographic system was consisting of a binary pump 200 series HPLC apparatus (Agilent Technologies, USA), coupled with an AB Sciex hybrid triple quadrupole mass spectrometer (AB Sciex, Canada) with a vacuum degasser, binary gradient pump and auto sampler. A Phenomenex Aqua-C18 column (2.0 mm × 50 mm, I.D. 5 mm) was used for the separation at a flow rate between 0.25 and 0.4 mL/min. The mobile phase was a gradient of 0.1% formic acid in water and 5 mM ammonium formate (solvent A) and 0.1% formic acid in acetonitrile and 5 mM ammonium formate (solvent B) with the following gradient programme: 0.01–8 min, 10% A to 90% B and hold for 2 min and back to 10% A in 0.1 min and re-equilibrated for 5 min. Total run time is 15 min.

The ESI-MS conditions were as follows: negative-ion mode; ionization voltage, -4.5 kV; drying and nebulizer gas is purified N_2_, total flow rate 34 L · min − 1; temperature, 500°C and pressure of nebulizer gas, 110 psi. Mass detection was performed in full scan mode in the range 100–1200 m/z.

The ESI-MS/MS conditions: collision gas is purified nitrogen gas. The collision energy is an average collision energy spread between 20 eV, 35 eV and 50 eV. Data analysis was acquired by AB Sciex Analyst 1.5 workstation and based on spectral MS/MS library match, with additional information from reference journal, and utilizing advanced chemometrics software data processing and interpretation with fragmentation prediction (ACD/Labs MS Fragmenter).

### Primary culture of human gingival fibroblast cells

The primary explant technique is a modified version described by Freshney [31]. Freshly extracted teeth with gingival tissues were collected from the Oral Surgery Department, Faculty of Dentistry, University of Malaya, Kuala Lumpur, Malaysia. The teeth were then placed in basic media of DMEM. Only tooth that was sound or healthy was selected for this procedure. The selected tooth was then washed with phosphate buffered saline and disinfected using penicillin/streptomycin solution for 2-3 min to kill any bacteria, and then rinsed with DMEM. The gingival tissue specimens were sliced into pieces, approximately 1×1 mm in size by using sterilized sharp scalpels. The tissues were then transferred into a 1.5 mL sterile centrifuge tube containing 1 mL of DMEM and penicillin/streptomycin solution (1:1 v/v) and gently resuspending them by pipetting up and down for a few seconds. The media were discarded, rinsed twice with DMEM and replaced with 1 mL of DMEM supplemented with 10% (v/v) fetal bovine serum (FBS).

The tissues were then transferred and carefully spread into the T25 flask coated with 0.1% gelatin. The tissues were incubated in a humid 5% CO_2_ incubator at 37°C to allow the attachment of the tissue to the surface of the flask. After one hour, an additional 500 μL of DMEM supplemented with 10% FBS was added into the flask, just enough to moisten the tissue. After 24 hours incubation, the medium was removed and gently replaced with 2 mL of DMEM supplemented with 10% FBS. The medium was replaced with a fresh medium every 3 days until the cells reached 70-80% confluence.

### Cytoprotective activity

**Figure 1:**
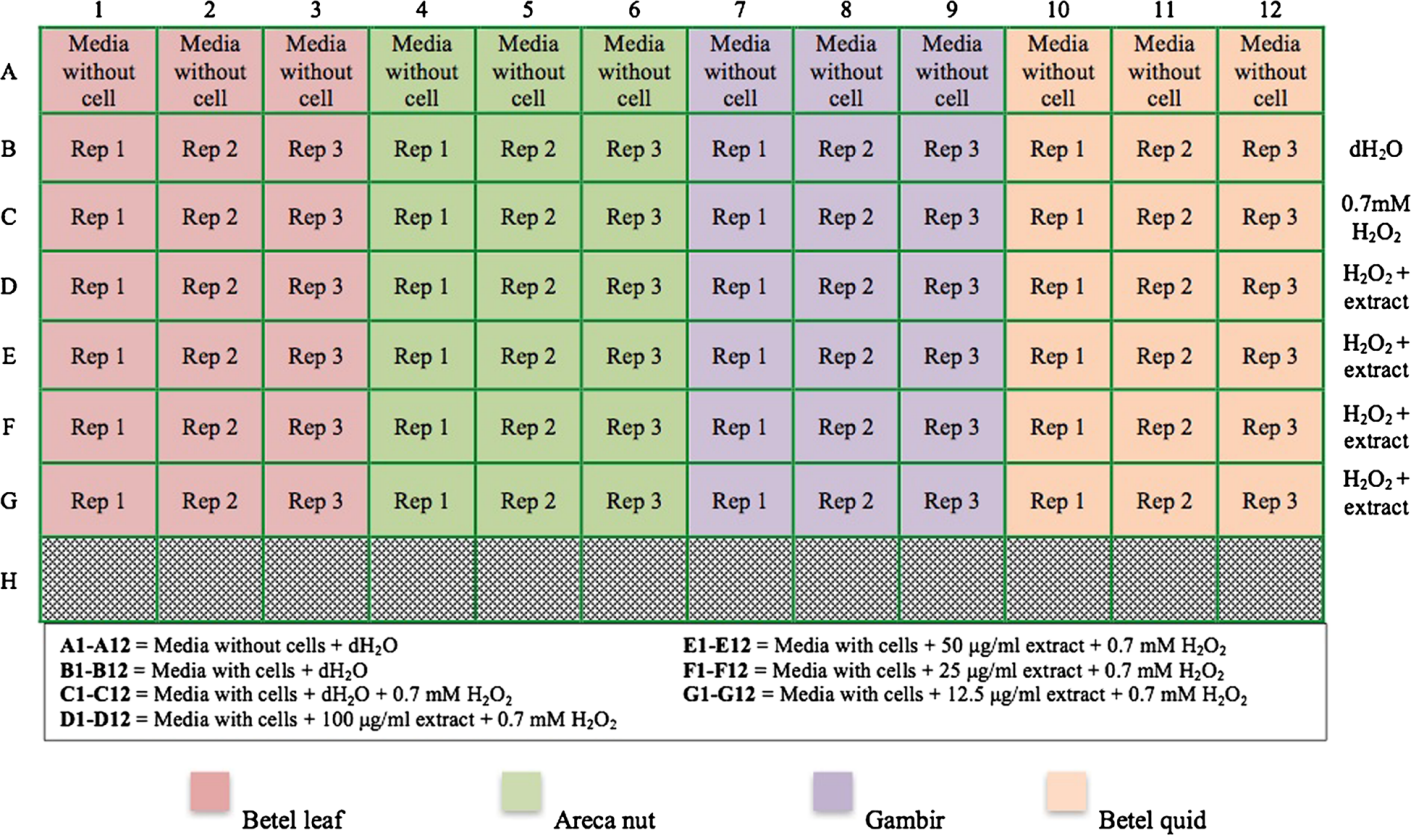
**Layout of the 96-well plate for cytoprotective analysis.** Different colours indicate different extract tested. Ascorbic acid was used as positive control.

100 μL of media (DMEM supplemented with 10% FBS) containing HGF cells at a density of 6×10^3^ cells/well were seeded in 96-well plates and incubated in a humid 5% CO_2_ incubator at 37°C for 8 hours (Figure 1). The media were then aspirated and replaced with fresh media containing extracts at different concentrations (12.5, 25, 50 and 100 μg/mL) and allowed to incubate for 16 hours. The media were discarded and rinsed with PBS. A media containing 0.7 mM of hydrogen peroxide was then added and incubated for 6 hours. The media were then discarded and 25 μL of freshly prepared MTT solution (5 mg/mL of PBS) was added and allowed to incubate for 4 hours. The supernatant was removed and the formazan blue crystals formed were dissolved in 150 μL of DMSO. The absorbance of the dissolved formazan blue cyrstal was measured at 570 nm using a microplate ELISA reader (Biotek U-quant, USA). The blank control referred as wells containing cells without the treatment of sample extract and hydrogen peroxide (deionized distilled water was used instead of the sample extract and hydrogen peroxide). For positive control, ascorbic acid was used. The percentage of cell viability was calculated with the following equation:

$$\mathrm{Cell}\;\mathrm{viability}\left(\%\right)=\left(A_{0}–A/A_{1}\right)\times 100\;\%$$

Where A was the absorbance of sample blank, A_1_ was the absorbance of the cells in the blank control and A_0_ was the absorbance of the cells (1) in the presence of the extract/ascorbic acid and (2) in the absence of extract after exposure with hydrogen peroxide. In the experiment, HGF used was from the fifth passage through eight passages.

### Statistical analysis

The experiments were repeated using three different sets of freshly prepared extracts and each of which was analyzed in triplicate and the result expressed as mean ± standard deviation (SD). For cytoprotective activity, the experiment was also analyzed in triplicate and expressed as mean ± standard deviation. Data were analyzed using SPSS statistical software, version 19.0 (SPSS Inc, Chicago, IL, U.S.A.). The tests were divided into two statistical tests, parametric and non-parametric tests. The parametric test includes one-way ANOVA and t-test, and the non-parametric test includes Kuskal Wallis test and Mann Whitney test. The *p* < 0.05 was considered significant whereas *p* < 0.01, highly significant. For correlation studies, Pearson’s correlation coefficient (r) was performed.

## Results

### Radical scavenging activity

Gambir exhibited the highest antioxidant activity by strongly inhibiting 50% of DPPH radicals at a lower concentration and was as potent as ascorbic acid (Table 1). However, betel quid with calcium hydroxide was unable to scavenge 50% of DPPH radicals even at the highest concentration tested (1 mg/mL). The IC_50_ of the extracts can be described in the following order: gambir > areca nut > betel quid > betel leaf > betel quid with calcium hydroxide.

### Ferric reducing activity

Similar pattern was observed with radical scavenging activity, ferric reducing activity was highest for the gambir extract and this was followed in descending order by the areca nut, betel quid, betel leaf and betel quid with calcium hydroxide (Table 1). In comparison with ascorbic acid, gambir is 3-folds lower in its ability to reduce the ferric ion.

### Inhibition on lipid peroxidation

All of the extracts demonstrated higher inhibition of lipid peroxidation compared to α-tocopherol at a concentration of 50 μg/mL after seven days of incubation (Table 1). The percentage of lipid peroxidation inhibition can be described in the following order: betel leaf > betel quid > betel quid with calcium hydroxide > areca nut > gambir. However, there was no significant difference in the inhibition activity among the extracts (*p* > 0.05).

### Total phenolic contents

It was found that the gambir extract has the highest TPC followed by areca nut (Table 1). The TPC of betel quid was 3-folds higher than the TPC of betel quid with calcium hydroxide. The TPC of betel quid with calcium hydroxide was the lowest. The TPC of the betel leaf was 1.8-folds lower than the TPC of betel quid but 1.7-folds higher than the TPC of betel quid with calcium hydroxide.

**Table 1:** Total phenolic and antioxidant activities of aqueous extract of plant materials

**Sample**	**TPC (μg TAE/mg)**	**DPPH (IC**_**50**_**) μg/mL**	**FRAP (μmol Fe(II)/mg)**	*Lipid peroxidation inhibition (%)
Betel leaf	77.2 ± 12.6	179.5 ± 93.1	357.3 ± 129.5	83.0 ± 0.2
Areca nut	858.8 ± 53.9	7.5 ± 0.5	4127.8 ± 192.8	78.8 ± 1.0
Gambir	1142.5 ± 106.8	6.4 ± 0.8	5717.8 ± 537.6	75.2 ± 1.2
Betel quid	140.0 ± 22.3	59.4 ± 4.4	1022.2 ± 235.7	82.7 ± 0.9
Betel quid + CaOH_2_	45.4 ± 3.7	Not detected	71.8 ± 29.8	82.2 ± 0.5
Positive control	-	4.8 ± 0.5^a^	16813.3 ± 131.1^a^	18.1 ± 2.8^b^

Values are expressed as means ± SD (n = 9). Values within each column were significantly difference at *p* < 0.05 (Kruskall Wallis test). Positive control: ^a^Ascorbic acid and ^b^α-tocopherol. *Sample tested at concentration 50 μg/mL after seven days of incubation.

### Analysis of phenolic compounds using LC-MS/MS

Table 2 shows the LC-MS/MS analysis showing the presence of eugenol in all of the betel leaf-based samples, which was highest in the betel leaf itself, followed by the betel quid and subsequently, the betel quid with calcium hydroxide. The major compound in betel leaf was *p*-hydroxybenzoic acid and in gambir was quinic acid. It was shown that betel quid has both *p*-hydroxybenzoic acid and quinic acid but in smaller amount. For the areca nut, the major compound was found to be catechin. Both *p*-coumaric acid and hydroxychavicol were present in the betel quid and betel quid with calcium hydroxide. The betel quid contained lower hydroxychavicol and higher *p*-coumaric acid than betel quid with calcium hydroxide.

**Table 2:** The composition of phenolic compounds in betel quid (with and without calcium hydroxide) and that of its ingredients

***t***_**R **_**(min)**	**MW (calculated mass)**	**M-H (observed mass)**	**Proposed compound**	**Area (%)**
**Betel leaf**				
1.454	138.03	137.07	*p*-hydroxybenzoic acid*	44.9
2.102	164.16	163.09	Eugenol	2.95
2.427	338.19	337.19	4-*p*-coumaroylquinic acid	1.6
**Areca nut**				
1.128	866	865.20	Procyanidin trimer	1.56
1.290	578	577.12	Procyanidin dimers (B1)	25.34
1.614	290.26	289.19	Catechin*	41.44
2.585	866	865.37	Procyanidin trimers	2.93
2.909	578	577.24	Procyanidin dimers (B2)	0.6
3.718	624	623.16	isorhamnetin 3-O-rutinoside	3.53
**Gambir**				
0.805	192.17	191.05	Quinic acid*	21.77
1.293	290.26	289.17	(+)-catechin	6.02
1.619	578.52	577.19	Procyanidin dimer (B1)	17.96
2.268	562	561.12	(epi)afzelechin-(epi)catechin	7.06
2.431	580.1	579.10	Proanythocyanidin dimer	12.16
2.593	578.52	577.15	Proanthocyanidin dimer	0.8
2.917	290.26	289.12	(-)-epicatechin	1.61
3.726	290.26	289.12	Catechin Isomer	5.34
5.505	578.52	577.11	Proanthocyanidin dimer	3.05
5.342	580.1	579.10	Proanthocyanidin dimer	3.07
6.28	610	609.19	Quercetine diglycoside	14.79
6.649	450.39	451.22	Cyanidin-3-O-glucoside	4.3
7.631	302.24	301.11	Quercetin	2.02
8.116	286.06	285.08	Kaempferol	0.05
**Betel quid**				
0.969	192.17	191.07	Quinic acid*	10.76
1.295	164	163.08	*p*-coumaric acid	4.55
2.426	164	163.05	Eugenol	3.89
2.587	134.18	133.02	Chavicol	1.26
3.071	138.03	137.03	*p*-hydroxybenzoic acid	1.52
6.460	150.174	149.08	hydroxychavicol	0.62
8.078	330	329.24	3,30-di-O-methyl ellagic acid	0.16
**Betel quid with calcium hydroxide**				
1.453	164.2	163.05	Eugenol	1.37
2.425	164.2	163.04	*p*-coumaric acid	1.61
7.098	150.174	149.07	Hydroxychavicol*	2.1

*Major compound in the aqueous extracts.

### Cytoprotective effects of the extracts on HGF cells exposed to hydrogen peroxide

**Figure 2:**
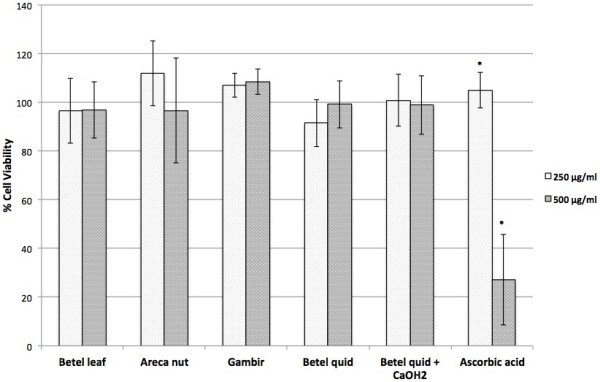
**Effects of extracts on human gingival fibroblast cells.** Cell viability was determined by MTT assay. Values are expressed as means ± SD (n = 3). *Significant difference at *p* < 0.05 (t-test).

Preliminary study by MTT assay showed that all of the extracts were not toxic up to a concentration of 500 μg/mL. Ascorbic acid was observed to be toxic at 500 μg/mL (Figure 2). Subsequent studies for cytoprotective effect used the extracts with concentration below 100 μg/mL.

**Figure 3:**
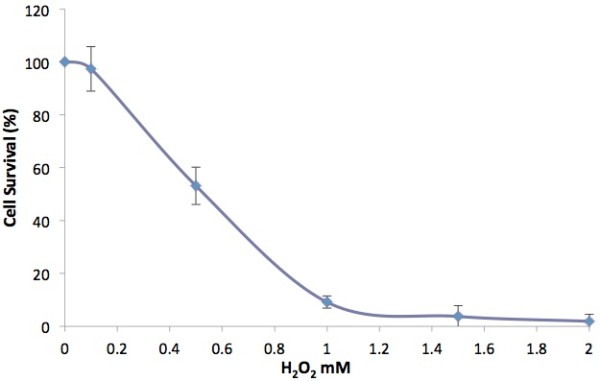
**Effects of hydrogen peroxide on human gingival fibroblast cells.** Cell viability was determined by MTT assay. Values are expressed as means ± SD (n = 9).

Figure 3 shows that the exposure of the cells to 0.5 mM hydrogen peroxide resulted in 53.1 ± 7.0% cell survival while the exposure with 1 mM hydrogen peroxide resulted in significant cell death with 9.2 ± 2.2% cell survival. Using this as a reference, the concentration of hydrogen peroxide that will be used to induce stress to the cells before treatment with the extract was 0.7 mM.

### Cytoprotective activity

**Figure 4:**
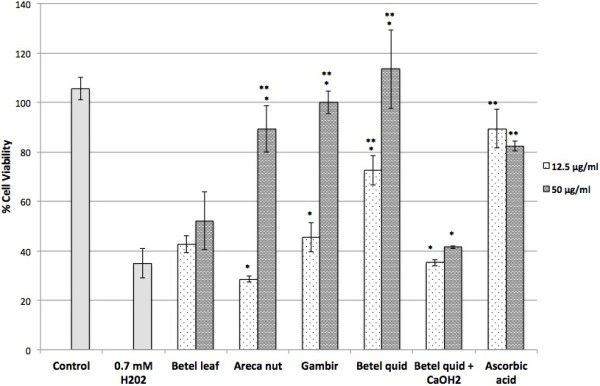
**Cytoprotective effects of the extracts against hydrogen peroxide-induced oxidative stress cells.** Values are expressed as means ± SD (n = 3). Ascorbic acid was used as positive control. *Significant difference within the same samples at *p* < 0.05 (t-test). **Significant difference when compared to H202 at *p* < 0.05 (t-test).

At 0.7 mM, hydrogen peroxide decreased cell viability to 34.9 ± 6.0% of that of the control (Figure 4). Areca nut, gambir and betel quid extracts exhibited strong cytoprotective effect at a concentration of 50 μg/mL with cell viability of 89.3 ± 9.4%, 100.1 ± 4.6% and 113.5 ± 15.9% respectively. This effect was comparable to that of ascorbic acid at a concentration of 50 μg/mL with cell viability of 82.4 ± 2.1%. The extracts of betel leaf and betel quid with calcium hydroxide at concentration 50 μg/mL; however, demonstrated slight cytoprotective effect against hydrogen peroxide-induced oxidative stress with 52.1 ± 11.7% and 41.5 ± 0.5% of cell viability respectively. The betel quid extract was also cytoprotective at a lower concentration of (12.5 μg/mL) with cell viability of 72.4 ± 5.9% while other extracts gave negligible cytoprotective effects (cells viability of 42.7 ± 3.4% for the betel leaf and 45.5 ± 5.9% for the gambir). Both the areca nut and betel quid with calcium hydroxide failed to protect the cells from hydrogen peroxide-induced stress at concentration of 12.5 μg/mL with cells viability of 28.5 ± 1.1% for the areca nut and 35.2 ± 1.3% for the betel quid with calcium hydroxide.

### Correlation of total phenolic content with antioxidant and cytoprotective activities

A positive and strong correlation (r) exists between TPC and DPPH radical scavenging and ferric reducing activity (Table 3). However a negative and strong correlation was observed between TPC and inhibition of lipid peroxidation activity (*p* < 0.01). The correlation between the TPC and cytoprotective activity was positive but less strong.

**Table 3:** Correlations between TPC with antioxidant and cytoprotective (CP) activities

**Assay**	**TPC vs 1/DPPH**	**TPC vs FRAP**	**TPC vs FTC**	**TPC vs CP**
r value	0.972	0.981	-0.951	0.682
*p* value	< 0.01	< 0.01	< 0.01	0.005

## Discussion

Betel quid is chewed in the oral cavity where it remains for some time, releasing its water-soluble components into the oral environment. Thus, a temperature of 37°C was set for the extraction using aqueous medium to emulate the condition in the oral cavity. In the DPPH and FRAP assays, it was shown that the gambir, one of the ingredients of betel quid exhibited a marginally better antioxidant activity than the areca nut. In addition, the DPPH scavenging activity of gambir is almost similar with ascorbic acid, indicating its powerful free radical scavenger. DPPH assay measures the ability of the extract to donate hydrogen to the DPPH radical resulting in bleaching of the DPPH solution. The greater the bleaching action, the higher is the antioxidant activity. The DPPH radical scavenging activity of the areca nut was comparable with what has been reported by Khan et al. [32].

The gambir extract also exhibited a strong antioxidant activity by reducing ferric ion (Fe^3+^) to ferrous (Fe^2+^) in FRAP assay. According to Alothman et al. [33], DPPH and FRAP assays share similar mechanism of reaction, that is to reduce certain radicals (e.g. ferric ion and DPPH radical). Thus the higher the scavenging DPPH-radical activity, the higher is the ferric reducing ability.

The DPPH radical scavenging and ferric reducing activities of betel quid containing calcium hydroxide were observed to be the lowest. This may be due to the alkalinity of the calcium hydroxide (the pH of betel quid with calcium hydroxide in aqueous solution was 7.69). Settharaksa et al. [34] have reported that an alkaline pH reduced total phenolic content and DPPH radical scavenging activity.

However, the extract of betel quid containing calcium hydroxide exhibited similar antioxidant activity with the other extracts in inhibiting the lipid peroxidation as measured in FTC assay. Moreover, all of the extracts demonstrated higher inhibitory effect towards the lipid peroxidation compared to α-tocopherol, indicating that the extracts are stronger lipid peroxidation inhibitor than the α-tocopherol. Previous study done by Maznah et al. [35] have reported that their extracts had the highest antioxidant activity compared with α-tocopherol, and the same finding was also reported by Huda-Faujan et al*.*[36], Hu et al. [37] and Aqil et al*.*[38].

The total phenolic content (TPC) in gambir was found to be the highest compared with the other extracts. This may be due to its high content of phenolic compounds as determined by the LC-MS/MS (Table 2). Other studies have reported that the gambir contains quinic acid [39], catechin [12,40], epicatechin [41], and procyanidin dimers B-1 [42]. The betel quid containing calcium hydroxide has low content of phenolics which corresponds to its low TPC. The free radical scavenging and ferric reducing activities of the extracts were strongly and positively correlated with its total phenolic content due to similar mechanisms involved in the assays. Folin-Ciocalteu method in TPC assay is based on the mechanism of oxidation and reduction [43,44], which is the same with the mechanism exhibited by free radical scavenging and ferric reducing activities. Thus, the higher the TPC, the stronger is the DPPH radical scavenging and ferric reducing activity. However, the negative correlation between lipid peroxidation inhibition activity and its total phenolic content may be related to the fact that only specific phenolic structures are involved in the lipid peroxidation assay [45]. This may explain why extracts with lowest TPC could strongly inhibit the lipid peroxidation.

Basically, the entire oral environment is likely to be exposed to the effects of the extract from betel quid chewing. The quid may be in close contact with the oral mucosa for prolonged periods and sometimes the chewers like to retain the quid in the mouth in the buccal areas that have been reported to correspond to the site of oral injuries [46]. As mentioned by Jeng et al. [47], oral keratinocytes and fibroblasts appear to be the major target cells attacked by the betel quid ingredients. Thus human gingival fibroblast (HGF) was selected to investigate the cytoprotective activity of betel quid (and its respective ingredients) against hydrogen peroxide-induced stress.

The positive correlation between the cytoprotective activity and TPC of the extracts (Table 3) indicates that the extracts with high phenolic content, demonstrate cytoprotective capability. This includes the extracts of areca nut, gambir and betel quid that were strongly effective at concentration of 50 μg/mL. Cui et al*.*[48] have reported that the extract containing polyphenolic that showed strong antioxidant activity demonstrated the most potent cytoprotective effects against hydrogen peroxide-induced cell damage. It was also suggested that the highest cytoprotective activity demonstrated by betel quid may be due to synergy effects, which is the interaction of areca nut, gambir and betel leaf to produce a cytoprotective effect greater than their individual effects. The betel quid with calcium hydroxide extracts showed lowest cytoprotective effects and this corresponds to its lowest TPC and there was no radical scavenging activity detected at concentration below than 1 mg/mL.

The possible phenolic compounds related to antioxidant and cytoprotective activities of the extracts are shown in Table 2. The major compound found in gambir was quinic acid. Quinic acid is derived from caffeic acid, a major class of hydroxycinnamic acid. Caffeic acid is present naturally in food as an ester with quinic acid, known as the chlorogenic acid (4-*p*-coumaroylquinic acid) [49]. Both chlorogenic acid and caffeic acid are antioxidants, protecting low-density lipoprotein (LDL) from oxidation and thus may play a role in preventing many age-related-diseases [50]. Pero [39] has reported the presence of natural ester of quinic acid in the aqueous extract of *Uncaria* species (gambir), and the importance of quinic acid as anti-viral, anti-oxidant and anti-aging as well as neurogenerative prevention with health benefit. The quinic acid was also found to be the major compound in betel quid.

The gambir aqueous extract also contains quercetin, which has been reported to have strong antioxidant properties [51]. Quercetin chelates metals, scavenges oxygen free radicals [52] and inhibits lipid peroxidation [53]. In normal cells, quercetin may protect the cells from necrosis and apoptosis [54]. However, it has been found to be more toxic towards malignant cells than normal cells [55,56]. It specifically inhibits cell proliferation and induces apoptosis in many cancer cell types [57].

The areca nut contains monomeric, dimeric and trimeric procyanidins that are known to be strong antioxidants. These findings were similar with the one that has been reported by Nonaka et al. [58]. It has been reported that the proanthocyanidins have antitumor, immunomodulatory, antiallergic, antioxidative, chemopreventive, and anti-inflammatory activities [59,60]. Catechin, a monomer of procyanidin may also have an influence on the antioxidant and cytoprotective properties in areca nut and gambir. The catechin, which is abundant in areca nut, is said to have a high ability to scavenge free radicals [12].

*p*-Hydroxybenzoic acid is the major compound in the extracts of betel leaf and betel quid. It is an antioxidant and has been used as food preservative. *p*-Hydroxybenzoic, a white crystalline powder is slightly soluble in water. Quinic acid is absent in the betel leaf extract and this may explain for the difference in the antioxidant properties between betel leaf and betel quid. Therefore, the high antioxidant properties demonstrated by betel quid in comparison with betel leaf may be related to the presence of quinic acid.

Hydroxychavicol, eugenol and *p*-coumaric acid are found in both betel quid and betel quid with calcium hydroxide. *p*-Coumaric acid is an antioxidant, which is a by-product of chlorogenic acid breakdown. *p*-Coumaric may lower serum cholesterol level, constraint low-density lipoprotein (LDL) from being oxidized, and maintain the high-density lipoprotein (HDL) level in addition to its antioxidant properties. The level of *p*-coumaric acid was found to be higher in betel quid than in betel quid with calcium hydroxide. This may explain for the difference in the antioxidant properties between the two.

Hydroxychavicol and eugenol are phenolic compounds commonly found in betel leaves. Hydroxychavicol is said to possess antibacterial [61,62], antioxidant and anticarcinogenic activities [63] whereas eugenol has been used as a local anesthetic for toothaches [64]. In addition, Baliga et al. [65] reported that the eugenol and hydroxychavicol in betel leaf are excellent antimutagens and are applicable against wide ranges of environmental carcinogens in both prokaryotes and eukaryotes.

Many studies have reported that hydroxychavicol is a major phenolic compound in the aqueous extract of betel leaves [1,20,66]. However our study has shown the absence of hydroxychavicol in the extract of betel leaf. This could be due to different procedure used in the extraction. For instance, the betel leaves were extracted in boiling water [1,66]. According to Pin et al. [20], raising the temperature to 60°C during extraction could increase the diffusivity and solubility of hydroxychavicol. However the presence of hydroxychavicol in the extract of betel quid and betel quid with calcium hydroxide suggests that the inclusion of areca nut and gambir in the quid may help the diffusivity of hydroxychavicol.

The alkaline condition of betel quid containing slake lime may also contribute to the diffusivity of hydroxychavicol. A significant amount of eugenol in the betel quid could be changed in the presence of calcium hydroxide, hence explaining for its low content in betel quid with calcium hydroxide. Sakano et al., [67] have reported that eugenol can be metabolized into hydroxychavicol in certain conditions. Thus it may suggest that eugenol from betel quid has been metabolized into hydroxychavicol, which resulted in the higher amount of hydroxychavicol in the betel quid containing calcium hydroxide.

Catechin and procyanidin, which are abundant in areca nut and gambir extract, were however absent in the extract of betel quid and betel quid with calcium hydroxide. Several studies have indicated that the stability of the catechin is affected by pH of the extracts. Zhu et al. [68] have reported that the catechin in green tea with pH above 8 are particularly unstable and quickly degrades but appears to be very stable at pH below 4. Su et al*.*[69] have also mentioned that the stability of catechins is pH-dependent. In both alkaline and neutral solutions, it is unstable as it decomposes rapidly but in acidic solution, relatively stable.

In this study, the pH for betel quid was neutral (6.90) and betel quid with calcium hydroxide was slightly alkaline (pH = 7.96) which explains for the absence of catechins in both the extracts. The absence of *p*-hydroxybenzoic acid and quinic acid in betel quid with calcium hydroxide also could be due to the alkaline nature of the extract. It may suggest that pH may give significant impact on the stability of phenolic compounds such as *p*-hydroxybenzoic acid, quinic acid and catechin.

## Conclusions

The total phenolic (TPC) content has an influence in radical scavenging, ferric reducing and cytoprotective activity of the extracts. The TPC as well as antioxidant and cytoprotective activities of betel quid (which is greater than betel quid containing calcium hydroxide) maybe associated with the presence of quinic acid. Further research should be carried out to identify and isolate the active compounds of *Areca catechu, Uncaria gambir* and *Piper betle* and perhaps betel quid that could be used as alternative sources of antioxidants for therapeutic purposes in oral healthcare.

## Competing interests

The authors declare that they have no competing interest.

## Authors’ contributions

NN designated the study, performed all the experiments, statistical analysis and drafted the manuscript. TN has been involved with interpretation of data and LCMS analysis. ZHAR has been involved with the preliminary investigation and revision of the manuscript for intellectual content. All authors read and approved the final manuscript.

## Pre-publication history

The pre-publication history for this paper can be accessed here:

http://www.biomedcentral.com/1472-6882/13/351/prepub
